# Microwave-Assisted Propolis Extract Attenuates Oxidative-Stress- and Replicative Senescence via NRF2 and Wnt/β-Catenin–TERT Activation in Human Dermal Fibroblasts

**DOI:** 10.3390/antiox15030395

**Published:** 2026-03-20

**Authors:** Seoungwoo Shin, Youngsu Jang, Kyungeun Jeon, Ji Yun Kim, De-Hun Ryu, Eunae Cho, Hyerin Yeo, Nae Gyu Kang, Deokhoon Park, Eunsun Jung

**Affiliations:** 1 BioSpectrum Life Science Institute, 767, Sinsu-ro, Yongin-si 16827, Republic of Korea; biost@biospectrum.com (S.S.); biogc@biospectrum.com (Y.J.); bioyd@biospectrum.com (K.J.); biosc@biospectrum.com (D.-H.R.); biozr@biospectrum.com (E.C.); pdh@biospectrum.com (D.P.); 2 Research and Development Center, LG Household & Healthcare (LG H&H), 70, Magokjoongang 10-ro, Seoul 07795, Republic of Korea; jykim49@lghnh.com (J.Y.K.); coramdeo@lghnh.com (H.Y.); ngkang@lghnh.com (N.G.K.)

**Keywords:** propolis, microwave-assisted extraction, fibroblast senescence, oxidative stress, NRF2, Wnt/β-catenin signaling, skin aging

## Abstract

Skin aging is characterized by fibroblast senescence, extracellular matrix (ECM) degradation, and impaired wound healing, driven by oxidative stress and telomere dysfunction. Here, we investigated the anti-aging effects of a standardized microwave-assisted propolis extract (MAPE) in both H_2_O_2_-induced and replicative senescence models of human dermal fibroblasts (HDFs). MAPE significantly reduced reactive oxygen species (ROS) accumulation and enhanced antioxidant gene expression (*NQO1*, *GCLM*), indicating activation of NRF2-dependent defense pathways. It suppressed senescence markers (*CDKN2A*, *CDKN1A*, *IL6*), decreased SA-β-gal activity, and attenuated inflammaging. Moreover, MAPE inhibited *MMP1* expression, restored *COL1A1*, and improved fibroblast wound closure, thereby maintaining ECM homeostasis. Importantly, MAPE modulated Wnt/β-catenin signaling by upregulating *WNT3A* and *LEF1* while suppressing *DKK1*, and increased *TERT* expression, suggesting involvement of telomerase-related regulatory pathways. These effects resembled those of CHIR99021, a canonical Wnt activator, while providing additional antioxidant protection. Together, our findings suggest that MAPE is a propolis-derived bioactive ingredient that counteracts fibroblast senescence through coordinated modulation of NRF2 and Wnt/β-catenin–TERT signaling pathways, supporting its potential as a cosmeceutical ingredient for mitigating skin aging.

## 1. Introduction

Skin aging is driven by both intrinsic and extrinsic factors, including telomere shortening, oxidative stress, and chronic inflammation [1]. Dermal fibroblasts, the major producers of extracellular matrix (ECM), progressively lose proliferative and regenerative capacity with age. Senescent fibroblasts display elevated expression of cyclin-dependent kinase inhibitors p16INK4a (encoded by *CDKN2A*) and p21CIP1 (encoded by *CDKN1A*), increased secretion of pro-inflammatory cytokines such as IL-6, and enhanced activity of senescence-associated β-galactosidase (SA-β-gal). These changes contribute to ECM imbalance, characterized by increased MMP-1 expression, reduced collagen synthesis, and impaired wound healing capacity, thereby accelerating dermal aging and wrinkle formation [2,3].

At the molecular level, oxidative stress is a central driver of cellular senescence. Excessive ROS accumulation activates DNA damage responses, senescence signaling, and the senescence-associated secretory phenotype (SASP), which further propagates inflammaging and tissue dysfunction [4]. Nuclear factor erythroid 2-related factor 2 (NRF2) is a key regulator of cellular antioxidant defense and maintains intracellular redox homeostasis by inducing cytoprotective genes such as NAD(P)H quinone dehydrogenase 1 (*NQO1*) and glutamate–cysteine ligase modifier subunit (*GCLM*) [5]. In dermal fibroblasts, maintenance of redox balance is particularly critical because oxidative stress directly promotes fibroblast senescence, matrix degradation, and impaired tissue repair during skin aging. Importantly, NRF2 activity has been reported to decline with age, resulting in impaired antioxidant defenses and increased vulnerability of dermal fibroblasts to oxidative stress [6,7]. Activation of NRF2 signaling has therefore been proposed as a protective mechanism that may mitigate oxidative stress-induced fibroblast dysfunction and help preserve dermal homeostasis. However, despite the recognized importance of oxidative stress in fibroblast aging, it remains unclear whether activation of the NRF2 pathway is sufficient to counteract established fibroblast senescence and restore dermal ECM homeostasis, particularly through modulation by natural bioactive compounds.

In parallel, the Wnt/β-catenin signaling pathway plays a pivotal role in regulating fibroblast proliferation, migration, and ECM synthesis. Canonical Wnt signaling stabilizes β-catenin, enabling its nuclear translocation and transcriptional activation of target genes such as *LEF1* and *COL1A1*, which are critical for collagen deposition and dermal homeostasis [8,9]. Conversely, suppression of Wnt signaling, mediated by inhibitors such as DKK1 or age-related decline in Wnt ligands, has been implicated in impaired fibroblast function, skin thinning, and delayed wound healing [10]. Age-related reduction in Wnt signaling contributes to decreased collagen synthesis and progressive ECM degradation, ultimately leading to dermal atrophy and impaired tissue repair. Importantly, Wnt signaling is also linked to the regulation of telomerase (*TERT*) expression, which may contribute to the maintenance of replicative capacity and tissue homeostasis [11]. Therefore, simultaneous activation of antioxidant defenses (NRF2) and senescence-modulating pathways (Wnt/β-catenin–TERT) may represent a promising strategy for mitigating skin aging.

Propolis, a resinous bee product rich in flavonoids, phenolic acids, and terpenoids, has long been recognized for its antioxidant, anti-inflammatory, and regenerative properties [12,13]. Several studies have reported that propolis extracts protect keratinocytes and fibroblasts from oxidative damage [14,15], enhance collagen synthesis, and accelerate wound healing [16]. Brazilian green propolis, for instance, was shown to suppress UVB-induced oxidative stress and inflammation in skin, thereby mitigating photoaging [17]. Moreover, bioactive flavonoids in propolis, including caffeic acid phenethyl ester (CAPE), quercetin, and chrysin, are known to modulate redox-sensitive signaling cascades and improve cellular resilience [18,19]. However, whether propolis extracts can directly modulate Wnt/β-catenin–TERT-related pathways in fibroblast senescence remains largely unexplored.

Microwave-assisted extraction (MAE) has emerged as an efficient technique for recovering bioactive compounds from natural products. Compared with conventional extraction methods, MAE offers several advantages, including accelerated mass transfer, reduced extraction time, and improved extraction efficiency, which enhance the recovery of phenolic and flavonoid compounds [20,21]. In addition, the use of polyol solvents such as butylene glycol provides a mild and cosmetically compatible extraction system that facilitates the direct incorporation of botanical extracts into topical formulations without solvent-removal steps.

Based on these considerations, MAPE (microwave-assisted propolis extract) was prepared from Jeju-derived propolis using a microwave-assisted butylene glycol extraction process designed to maximize the recovery of bioactive phenolic constituents.

In this study, we investigated the biological effects of MAPE in H_2_O_2_-induced and replicative senescence models of human dermal fibroblasts. We evaluated its impact on oxidative stress, senescence-associated markers, ECM-related gene expression, wound-healing capacity, and signaling pathways associated with NRF2 and Wnt/β-catenin–TERT regulation. In addition, its activity was compared with CHIR99021 (CHIR), a canonical Wnt activator, to better interpret the signaling responses observed in fibroblasts.

## 2. Materials and Methods

### 2.1. Materials and Preparation of Propolis Extract

Raw Propolis Material: Raw propolis was obtained from the Jeju Honey Agricultural Association Corporation (Jeju-si, Republic of Korea). All samples were stored in a desiccated state at 4 °C until further processing.

Microwave-Assisted Extraction (MAE): MAPE, a standardized Jeju propolis extract, was prepared by microwave-assisted extraction to maximize the recovery of bioactive polyphenols while minimizing thermal degradation. Ground propolis (25 g) was mixed with butylene glycol at a solvent-to-solid ratio of 20:1 (*w*/*w*) in a borosilicate beaker. The mixture was subjected to microwave irradiation using a microwave system (LG Electronics, Seoul, Republic of Korea) at a power of 1000 W for 30 s, followed by a 30-s cooling interval. This heating–cooling cycle was repeated seven times (total microwave irradiation time: 210 s). During microwave irradiation, the extraction temperature was monitored and reached approximately 150 °C. After completion of the extraction, the mixture was allowed to cool to room temperature and was then filtered through qualitative filter paper (Whatman No. 1; Cytiva, Marlborough, MA, USA) to obtain the liquid extract, designated as MAPE.

Phytochemical Profiling and Chemical Fingerprinting (HPLC Analysis): High-performance liquid chromatography (HPLC) with photodiode array (PDA) detection was performed to compare the chemical profiles of the different propolis extracts, including MAPE. To establish a comprehensive phytochemical fingerprint of the selected extract, MAPE was screened for 20 common phenolic compounds (gallic acid, D-(-)-Salicin, protocatechuic acid, scopolin, chlorogenic acid, puerarin, caffeic acid, vanillin, *p*-coumaric acid, ferulic acid, rutin hydrate, narirutin, hesperidin, rosmarinic acid, myricetin, quercetin, (±)-naringenin, apigenin, kaempferol, and formononetin). In addition, three representative propolis flavonoids—chrysin, pinocembrin, and galangin—were selected as marker compounds for extract standardization, and their contents were quantified. The analysis was performed using a Waters 2695 Separation Module equipped with a Waters 2996 PDA Detector (Waters Corporation, Milford, MA, USA). Chromatographic separation was achieved on a Luna C18 column (Phenomenex, Torrance, CA, USA) (4.6 mm × 250 mm, 5 μm) maintained at room temperature. The mobile phase consisted of solvent A (0.1 trifluoroacetic acid in water) and solvent B (acetonitrile), delivered at a flow rate of 1.0 mL/min. The gradient elution program was as follows: 0–50 min, 38% B; 50–52 min, linear increase to 100% B; 52–55 min, 100% B; 55–58 min, linear decrease to 38% B; 58–70 min, 38% B. The injection volume was 10 μL, and detection was performed at a wavelength of 280 nm. Three independent batches of MAPE were analyzed to confirm the reproducibility of the extraction process. Key compounds were identified by comparing their UV spectra (200–400 nm) and retention times with those of the corresponding standards. Quantification was performed using calibration curves generated from the standards.

Comparative Extraction Screening: To compare the efficiency of different extraction approaches, propolis was extracted using several solvents and extraction conditions prior to microwave-assisted extraction. Conventional thermal extraction was performed using ethanol, dipropylene glycol, water, and butylene glycol at 80 °C for 3 h with a solvent-to-solid ratio of 20:1 (*w*/*w*). The extraction efficiency of these methods was evaluated by determining total phenolic content (TPC). Microwave-assisted butylene glycol extraction (MAPE) showed the highest phenolic recovery and was therefore selected for subsequent phytochemical characterization and biological evaluation. Total phenolic content (TPC) was determined using the Folin–Ciocalteu colorimetric assay and expressed as gallic acid equivalents (GAE) [22].

### 2.2. Cell Culture

Primary human neonatal dermal fibroblasts (HDFs; PCS-201-010, ATCC, Manassas, VA, USA) were cultured in Dulbecco’s Modified Eagle’s Medium (DMEM; Welgene, Daegu, Republic of Korea) supplemented with 10% fetal bovine serum and penicillin/streptomycin (100 U/mL and 50 μg/mL, respectively) at 37 °C in a humidified atmosphere containing 5% CO_2_. Oxidative stress-induced senescence was generated by continuously exposing HDFs (passage ≤ 15) to hydrogen peroxide (H_2_O_2_, 300 μM) for 5 days, and HDFs at the same passage without H_2_O_2_ exposure were used as controls. Replicative senescent fibroblasts were obtained by serial subculture until passage ≥ 40, at which point cells exhibited typical morphological and proliferative features of senescence. All experiments were performed immediately after completion of the 5 day H_2_O_2_ exposure or upon reaching the designated passage number.

### 2.3. Cell Viability Assay

Cell viability was evaluated using an MTT assay. Human dermal fibroblasts were seeded in 6-well plates at a density of 1 × 10^5^ cells per well and treated with MAPE (0.002–0.02%) for 5 days under the same replicative senescence conditions used for the SA-β-gal assay. After treatment, MTT solution was added and incubated for 3 h at 37 °C. The resulting formazan crystals were dissolved in DMSO, and absorbance was measured at 570 nm using a Epoch microplate reader (BioTek Instruments, Winooski, VT, USA).

### 2.4. RNA Isolation and Quantitative Real-Time RT–PCR

Total RNA was isolated with the MiniBEST Universal RNA Extraction Kit (9767A; Takara Bio, Shiga, Japan) according to the supplier’s protocol. First-strand cDNA was generated from 1 µg total RNA using the Applied Biosystems™ High-Capacity cDNA Reverse Transcription Kit (Thermo Fisher Scientific, Waltham, MA, USA) following the manufacturer’s instructions. Quantitative real-time PCR was carried out on a CronoSTAR™ 96 Real-Time PCR system (Takara Bio, Kusatsu, Japan). Gene-specific primer sequences are listed in Table 1.

### 2.5. SA-β-Gal Staining Assay

Human dermal fibroblasts (HDFs) were seeded at 1 × 10^5^ cells per well in 6-well plates and incubated with test materials for 5 days. Senescence-associated β-galactosidase (SA-β-gal) activity was visualized using a Senescence β-Galactosidase Staining Kit (Cell Signaling Technology, Danvers, MA, USA) following the manufacturer’s instructions [23]; cells positive for SA-β-gal appeared blue after overnight incubation with the staining solution. After overnight staining, blue-stained cells were imaged at ×100 magnification, and staining intensity was quantified as integrated density (IntDen) using ImageJ (Fiji, version 1.54p; NIH, Bethesda, MD, USA). For fluorescence-based detection of β-galactosidase activity, cells were labeled with SPiDER-βGal (Dojindo, Kumamoto, Japan) for 15 min at 37 °C, rinsed, and imaged by fluorescence microscopy under identical acquisition settings; the proportion of fluorescence-positive cells was determined from the acquired images.

### 2.6. Immunofluorescence Staining

Human dermal fibroblasts (HDFs) were seeded at 1 × 10^5^ cells per well in 6-well plates and incubated with test materials for 5 days. Cells were fixed in 4% formaldehyde for 15 min at room temperature, then blocked in 5% bovine serum albumin with 0.3% Tween-20 in PBS for 1 h. Primary antibodies against p21CIP1 and β-catenin (typical 1:500 dilution; CST, Danvers, MA, USA) were applied overnight at 4 °C, followed by species-appropriate Alexa Fluor-conjugated secondary antibodies (typical 1:1000 dilution; Invitrogen, Carlsbad, CA, USA). Nuclei were counterstained with Hoechst 33342 (1:10,000; 10 min, room temperature), and fluorescence was acquired using a K1-Fluo confocal microscope (Nanoscope Systems, Daejeon, Republic of Korea). Acquisition parameters were kept constant for all groups.

### 2.7. Intracellular ROS Measurement

Intracellular ROS were assessed using 2′,7′-dichlorodihydrofluorescein diacetate (H_2_DCFDA, Invitrogen, Thermo Fisher Scientific, Waltham, MA, USA) [24]. Cells were incubated with 5 µM H_2_DCFDA for 30 min at 37 °C, rinsed with PBS, and imaged under identical acquisition settings using a confocal microscope. Fluorescence was quantified in ImageJ/Fiji and expressed as relative fluorescence intensity.

### 2.8. Enzyme-Linked Immunosorbent Assay (ELISA)

Interleukin-6 (IL-6) levels in culture supernatants were quantified using a Human IL-6 Quantikine ELISA kit (R&D Systems, Minneapolis, MN, USA) according to the manufacturer’s instructions. Human dermal fibroblasts (HDFs) were subjected to oxidative stress by continuous exposure to H_2_O_2_ (300 µM) for 5 days and co-treated with MAPE at 0.005%, 0.01%, or 0.02%. At the end of the treatment period, culture supernatants were collected and analyzed for IL-6.

### 2.9. Scratch Wound-Healing Assay

Normal human dermal fibroblasts (HDFs; passages ≤10) were cultured to confluence (≈95–100%) in 6-well plates and switched to DMEM containing 1% FBS on the assay day. A linear wound was created across the cell monolayer using a sterile 200 µL pipette tip, and detached cells were removed by two PBS rinses. After scratching, cells were incubated with assay medium (DMEM with 1% FBS) containing MAPE (0.005, 0.01, or 0.02% *w*/*v*), or EGCG (10 µM) for 30 min, followed by the addition of H_2_O_2_ (300 µM) to induce oxidative impairment of cell migration. Corresponding vehicle-only wells (without H_2_O_2_) served as untreated controls. Wound recovery was quantified by measuring the cell-covered area within a fixed region of interest (ROI) at 72 h using ImageJ software. The cell-covered area was segmented from background using the threshold function, and the pixel area occupied by cells was recorded.

The relative recovery (%) was calculated according to the following formula:

$$\mathrm{Relative}\;\mathrm{recovery}\;(\%)=\frac{A_{\mathrm{sample}}-A_{H_{2}O_{2}}}{A_{\mathrm{control}}-A_{H_{2}O_{2}}}\times 100$$

where

$A_{\mathrm{control}}$ = cell-covered area in the vehicle (no H_2_O_2_) group;

$A_{H_{2}O_{2}}$ = cell-covered area in the H_2_O_2_-only group;

$A_{\mathrm{sample}}$ = cell-covered area in the test sample group.

### 2.10. Determination of Population Doubling Time

Normal human dermal fibroblasts (HDFs) were cultured in DMEM containing 10% FBS and 1% penicillin–streptomycin at 37 °C in a humidified 5% CO_2_ incubator. At designated time points (3 or 7 days after seeding), cells were detached and viable cell numbers were quantified using an automated cell counter (LUNA-II™, Logos Biosystems, Anyang-si, Republic of Korea) with disposable counting slides according to the manufacturer’s instructions. Viable cell numbers reported by the instrument were used for calculations.

Population Doubling Level (PDL) and Population Doubling Time (PDT) were calculated according to the following equations:

$$PDL=\;\log_{2}\left(\frac{N}{N_{0}}\right),\;\;PDT(day)=\frac{t\;(day)}{PDL}$$

where $N_{0}$ and N denote the initial and harvested cell numbers, respectively, and t represents the incubation period in days.

### 2.11. Statistical Analysis

All data are expressed as the mean ± SD, as indicated. Statistical analysis was performed using one-way ANOVA followed by Dunnett’s multiple comparisons test, and differences were considered statistically significant at *p* < 0.05.

## 3. Results

### 3.1. Comparison of Extraction Processes for Propolis Extracts

BG (1,3-butylene glycol) is commonly used as an extraction solvent in cosmetic manufacturing because of its good water compatibility and lower irritancy compared with ethanol [25]. In the present study, we applied a BG-based extraction process to propolis and compared a conventional BG-based extraction with a microwave-assisted BG-based extraction and other solvent systems.

To establish a comprehensive chemical fingerprint for MAPE, we initially performed a screening of 20 common phenolic compounds (listed in Section 2.1). None of these standard phenolic compounds were detected in MAPE under the present analytical conditions, suggesting that the extract possesses a phytochemical profile distinct from the screened phenolic standards (Supplementary Table S1; Supplementary Figure S1). Instead, quantitative analysis focused on three representative propolis flavonoids—chrysin, pinocembrin, and galangin—which were used as marker compounds for extract standardization.

**Figure 1:**
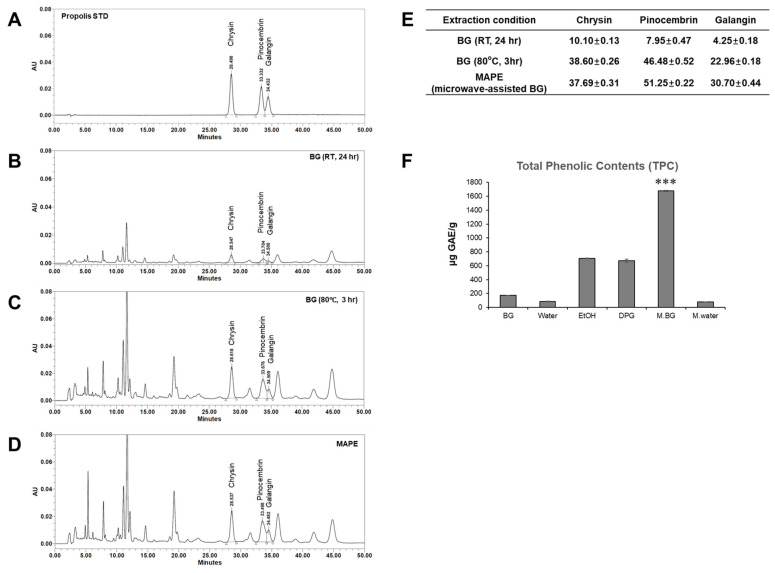
HPLC chromatograms and quantitative analysis of BG-based propolis extracts. (**A**) Propolis standard. (**B**–**D**) Chromatograms of BG extracts obtained at room temperature for 24 h, at 80 °C for 3 h, and by microwave-assisted BG extraction (MAPE). (**E**) Concentrations (µg/mL) of chrysin, pinocembrin and galangin in each BG-based propolis extract. (**F**) Total phenolic contents (TPC) of propolis extracts obtained under different extraction conditions. Data are presented as mean ± SD (*n* = 3). *** *p* < 0.001 vs. EtOH extract.

As shown in Figure 1A–D, HPLC analysis of three representative propolis flavonoids—chrysin, pinocembrin and galangin—showed that the microwave-assisted BG-based propolis extract (MAPE) contained slightly higher overall levels of these markers than the propolis extract obtained by BG extraction at 80 °C for 3 h, while both BG-based extracts yielded markedly higher levels than the propolis extract obtained by BG extraction at room temperature (24 h). In addition, when the total phenolic content was compared among six different extraction conditions (EtOH, DPG, water, microwaved water, BG without microwave and BG with microwave), the microwave-assisted BG process produced the highest value (1676.6 µg GAE/g), corresponding to a 2.3-fold increase over ethanol extraction and a 9.7-fold increase compared with BG extraction without microwave.

The superiority of the microwave-assisted BG process is likely attributable to rapid dielectric heating and increased internal pressure within the propolis matrix, which facilitate the release of phenolic compounds. Although the levels of the three quantified flavonoids were similar between the microwave-assisted and 80 °C BG extracts, the markedly higher total phenolic content suggests that microwave-assisted extraction improves the recovery of a broader range of phenolic constituents beyond these flavonoid markers.

On this basis, MAPE was used in the subsequent experiments to investigate its antioxidant and anti-aging effects in human dermal fibroblasts.

### 3.2. MAPE Alleviates H_2_O_2_-Induced Premature Senescence in Human Dermal Fibroblasts

**Figure 2:**
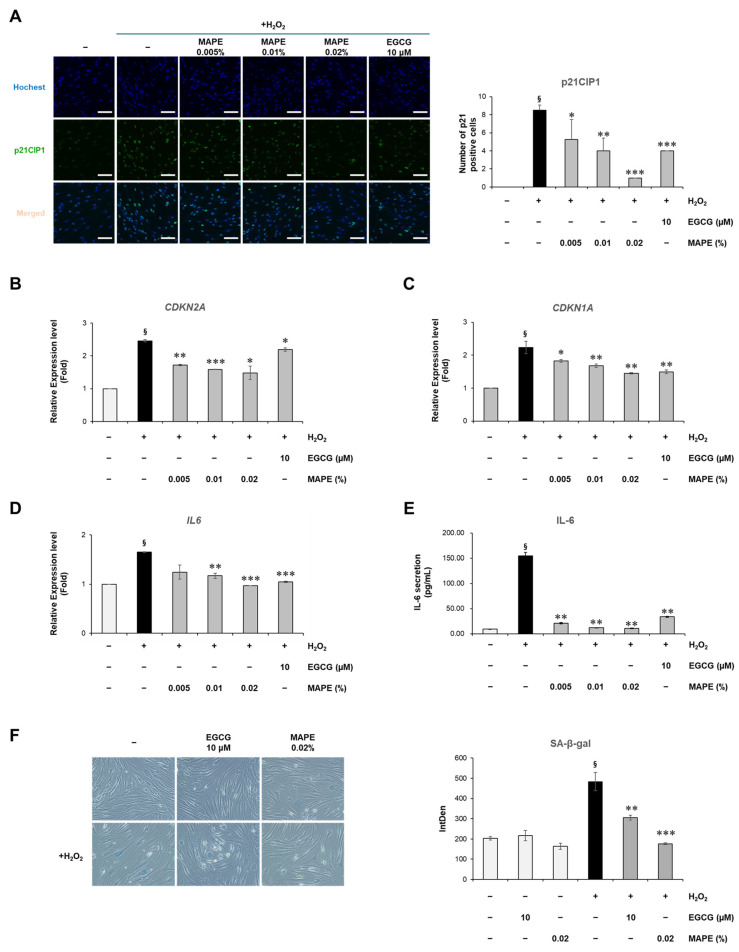
MAPE suppresses oxidative stress-induced senescence and SASP in human dermal fibroblasts. (**A**) Representative immunofluorescence images showing nuclear p21CIP1 expression (green) and nuclei (Hoechst, blue) in HDFs treated with H_2_O_2_ (300 µM) followed by MAPE (0.005–0.02%) or EGCG (10 µM) for 5 days. Quantification of p21CIP1-positive nuclei is shown on the right. (**B**,**C**) mRNA expression levels of *CDKN2A* and *CDKN1A* determined by qRT-PCR. (**D**) Relative *IL6* mRNA levels and (**E**) IL-6 secretion in culture medium quantified by ELISA. (**F**) Representative phase-contrast images showing SA-β-gal-positive cells (blue) in fibroblasts treated with H_2_O_2_ (300 µM) followed by EGCG (10 µM) or MAPE (0.02%) for 5 days. Quantification of SA-β-gal intensity is shown on the right. Data are presented as mean ± SD (*n* = 3). ^§^ *p* < 0.05 vs. untreated control; * *p* < 0.05, ** *p* < 0.01, *** *p* < 0.001 vs. H_2_O_2_-treated group. Scale bars: 100 µm.

To determine whether MAPE could suppress oxidative stress-induced cellular senescence, normal human dermal fibroblasts (HDFs) were treated with H_2_O_2_ in the presence or absence of increasing concentrations of MAPE (0.005–0.02%) or the reference antioxidant EGCG (10 µM), a well-established anti-aging compound. As shown in Figure 2A, H_2_O_2_ treatment markedly increased nuclear p21CIP1 staining and the proportion of p21CIP1-positive cells, indicating the onset of cell cycle arrest. MAPE significantly reduced p21CIP1 immunoreactivity in a dose-dependent manner, with the 0.02% treatment restoring the signal to nearly basal levels. Quantitative RT-PCR further confirmed a marked down-regulation of *CDKN2A* and *CDKN1A* expression (Figure 2B,C), both of which are canonical markers of cellular senescence.

In parallel, *IL6* mRNA expression and IL-6 protein secretion were markedly upregulated by oxidative stress but attenuated by MAPE (Figure 2D,E). IL-6 is a well-established SASP-associated cytokine [26,27]. ELISA analysis revealed that IL-6 secretion decreased to approximately 80% of the H_2_O_2_ control group following treatment with 0.02% MAPE, indicating potent anti-inflammatory and anti-SASP effects. Collectively, these findings indicate that MAPE effectively mitigates oxidative stress-induced fibroblast senescence, which is associated with suppression of *CDKN2A* and *CDKN1A* expression and reduced SASP cytokine production.

Consistent with the reduction in senescence-associated gene expression, MAPE also suppressed cellular senescence at the functional level. SA-β-galactosidase staining revealed a pronounced increase in senescence-positive fibroblasts after H_2_O_2_ exposure, characterized by flattened morphology and intense blue staining (Figure 2F). In contrast, cells treated with MAPE (0.02%) displayed fewer SA-β-gal-positive areas and retained a more elongated, spindle-like morphology similar to non-stressed controls. Quantitative image analysis confirmed that H_2_O_2_ treatment elevated β-gal activity intensity more than twofold compared to untreated controls, whereas MAPE reduced it by approximately 40% (*p* < 0.01 vs. H_2_O_2_ group). The magnitude of reduction was comparable to that observed with EGCG (10 µM), a well-established antioxidant reference.

These findings indicate that MAPE effectively alleviates oxidative stress-induced fibroblast senescence, restoring cellular morphology and reducing lysosomal β-gal activity associated with the senescent phenotype.

### 3.3. MAPE Suppresses Oxidative Stress and Restores Intracellular Redox Balance

**Figure 3:**
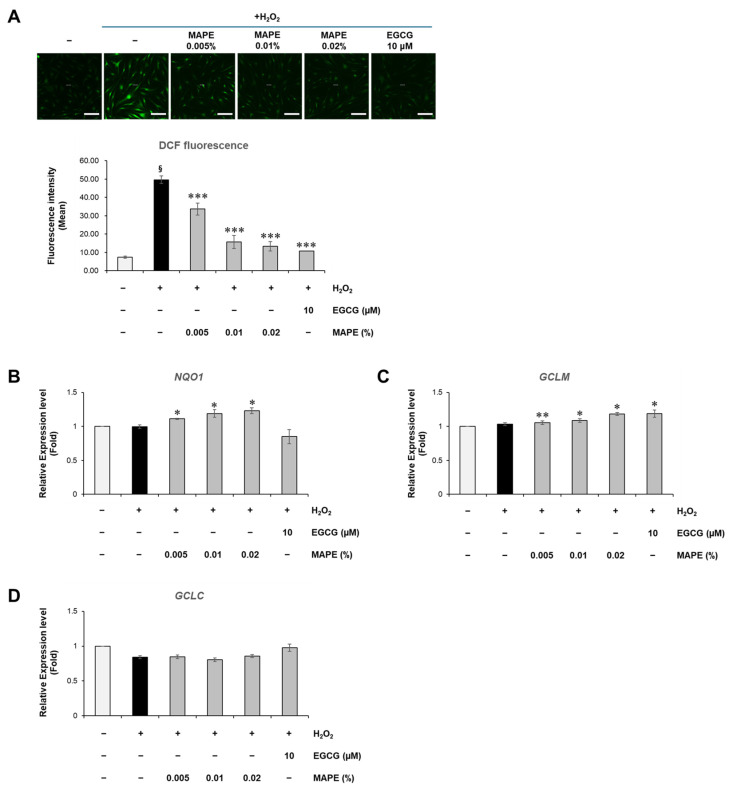
MAPE suppresses intracellular ROS accumulation and activates NRF2-related antioxidant genes in H_2_O_2_-treated fibroblasts. (**A**) Representative fluorescence images showing DCFH-DA-positive cells (green) and corresponding quantification of ROS intensity in HDFs exposed to H_2_O_2_ (300 µM) followed by treatment with MAPE (0.005–0.02%) or EGCG (10 µM) for 5 days. (**B**–**D**) Relative mRNA expression of NRF2 target genes *NQO1* (**B**), *GCLM* (**C**), and *GCLC* (**D**) determined by qRT-PCR. Data are presented as mean ± SD (*n* = 3). ^§^ *p* < 0.05 vs. untreated control; * *p* < 0.05, ** *p* < 0.01, *** *p* < 0.001 vs. H_2_O_2_-treated group. Scale bars: 200 µm.

After confirming that MAPE suppressed senescence-associated markers and SASP factors (Figure 2), we next examined whether these protective effects were accompanied by improvements in redox balance. To investigate whether the anti-senescent effect of MAPE was associated with attenuation of oxidative stress, intracellular ROS levels were quantified using the DCFH-DA probe (Figure 3A).

Fluorescence microscopy revealed that exposure to H_2_O_2_ triggered intense green fluorescence indicative of ROS generation, which was visibly attenuated in cells co-treated with MAPE. Quantitative analysis confirmed that H_2_O_2_ markedly increased ROS fluorescence intensity, indicating strong oxidative insult. However, treatment with MAPE significantly reduced ROS accumulation in a concentration-dependent manner, lowering fluorescence intensity by approximately 35–45% compared with H_2_O_2_-only groups. The antioxidant reference EGCG produced a comparable reduction.

These findings indicate that MAPE alleviates intracellular oxidative stress. This reduction in ROS prompted us to investigate whether MAPE acts merely as a direct radical scavenger or if it functions by boosting endogenous antioxidant defense systems.

### 3.4. MAPE Enhances Antioxidant Defense Through Upregulation of NRF2-Target Genes

To elucidate the molecular mechanism underlying the observed ROS reduction, we evaluated the expression of canonical NRF2-regulated antioxidant genes. As shown in Figure 3B–D, MAPE treatment resulted in a significant upregulation of *NQO1* and *GCLM* mRNA levels. Interestingly, *GCLC* expression showed no significant change, suggesting differential regulation of the glutathione synthesis subunits by MAPE. While H_2_O_2_ exposure alone induced a slight increase in *GCLM* expression consistent with a compensatory stress response, MAPE markedly potentiated this induction, elevating *NQO1* and *GCLM* levels significantly beyond the stress-induced baseline.

Collectively, these data indicate that the restoration of redox balance observed in Figure 3A is associated with activation of NRF2-dependent antioxidant pathways. By boosting the transcription of key cytoprotective enzymes, MAPE reinforces intracellular redox homeostasis, thereby equipping fibroblasts with enhanced resistance against oxidative insults.

### 3.5. MAPE Modulates ECM Remodeling by Downregulating MMP1 and Restoring COL1A1 Expression

**Figure 4:**
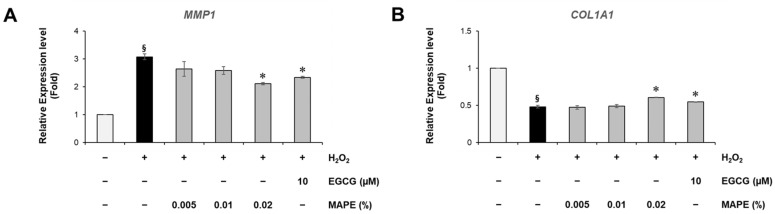
MAPE restores ECM homeostasis by suppressing *MMP1* and increasing *COL1A1* expression. qRT-PCR analysis of (**A**) *MMP1* and (**B**) *COL1A1* mRNA levels in HDFs following H_2_O_2_ (300 µM) exposure and treatment with MAPE (0.005–0.02%) or EGCG (10 µM). Data are presented as mean ± SD (*n* = 3). ^§^ *p* < 0.05 vs. untreated control; * *p* < 0.05, vs. H_2_O_2_-treated group.

Since oxidative stress and fibroblast senescence are closely associated with ECM degradation, we next assessed the effect of MAPE on matrix remodeling genes. Exposure to H_2_O_2_ markedly increased *MMP1* mRNA levels to approximately 3.08-fold of the untreated control, while reducing *COL1A1* expression to about 0.48-fold, a pattern consistent with increased collagen breakdown and impaired dermal structure. MAPE treatment reversed both trends in a dose-related manner (Figure 4). At 0.02%, MAPE significantly suppressed the H_2_O_2_-induced *MMP1* upregulation, inhibiting approximately 46.2% of the increase, and partially restored *COL1A1* expression, recovering about 26.9% of the H_2_O_2_-induced loss relative to the basal control level, with an efficacy comparable to the positive control EGCG (10 µM).

These results suggest that MAPE not only limits oxidative stress-induced ECM degradation but also helps restore collagen synthesis capacity, which may contribute to the preservation of dermal structural integrity under aging- or stress-related conditions.

### 3.6. MAPE Promotes Fibroblast Migration and Wound Closure Under Oxidative Conditions

**Figure 5:**
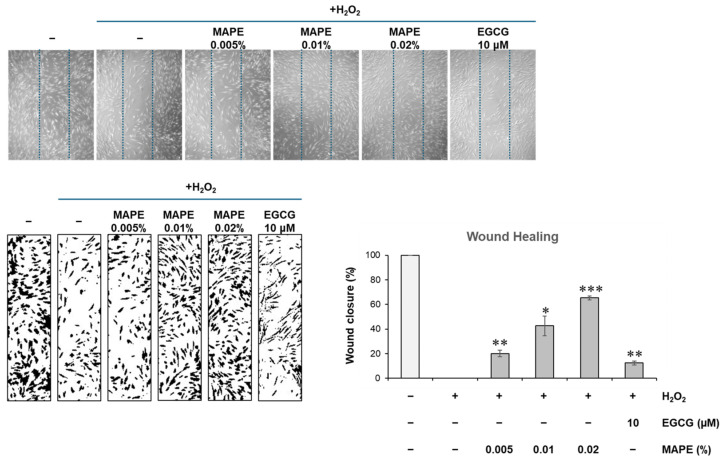
Representative phase-contrast images and quantification of wound closure in HDF monolayers treated with H_2_O_2_ (300 µM) and MAPE (0.005–0.02%) or EGCG (10 µM) for 72 h. Wound closure values were normalized to the H_2_O_2_-treated group (set to 0%) to represent the relative recovery of cell migration under oxidative stress conditions. Data are presented as mean ± SD (*n* = 3). * *p* < 0.05, ** *p* < 0.01, *** *p* < 0.001 vs. H_2_O_2_ group. Microscopic images were obtained at ×10 magnification.

To determine whether the molecular effects of MAPE translate into functional recovery of fibroblasts, we performed a scratch-wound assay under oxidative conditions (Figure 5). In H_2_O_2_-treated fibroblasts, migratory capacity was markedly impaired, resulting in negligible wound closure compared to the near-complete closure observed in untreated controls. However, co-treatment with MAPE significantly enhanced fibroblast migration in a dose-dependent manner. Notably, treatment with 0.02% MAPE achieved a wound closure rate of approximately 65.3%, calculated relative to the H_2_O_2_-treated group, representing a marked improvement compared to H_2_O_2_ alone, while EGCG (10 μM) produced only a modest effect. These findings indicate that MAPE effectively rescues fibroblast motility and wound-healing capacity compromised by oxidative stress.

Taken together, our data suggest that MAPE protects dermal fibroblasts from oxidative injury through complementary mechanisms: (i) attenuation of intracellular ROS accumulation via activation of the NRF2-dependent antioxidant response and (ii) preservation of ECM integrity and fibroblast functionality. This dual protection of redox balance and structural homeostasis may underlie the anti-senescent phenotype and improved dermal homeostasis observed in our in vitro skin-aging model.

### 3.7. MAPE Reactivates Wnt/β-Catenin Signaling Suppressed by Oxidative Stress

**Figure 6:**
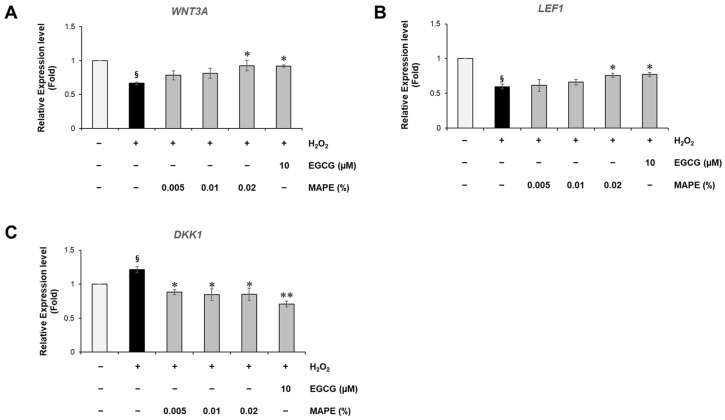
MAPE restores Wnt/β-catenin signaling suppressed by oxidative stress. (**A**,**B**) Relative mRNA expression levels of *WNT3A* (**A**) and *LEF1* (**B**) in HDFs exposed to H_2_O_2_ (300 µM) followed by treatment with MAPE (0.005–0.02%) or EGCG (10 µM), determined by qRT-PCR. (**C**) Relative mRNA expression of *DKK1*, a secreted Wnt antagonist. Data are presented as mean ± SD (*n* = 3). ^§^ *p* < 0.05 vs. untreated control; * *p* < 0.05, ** *p* < 0.01, vs. H_2_O_2_-treated group.

To further elucidate the molecular mechanism underlying the anti-senescent effects of MAPE, we examined genes involved in the Wnt/β-catenin pathway, which is essential for fibroblast proliferation, collagen synthesis, and tissue repair [6,7,8,9]. In H_2_O_2_-treated fibroblasts, the expression of *WNT3A* and *LEF1* was markedly reduced, indicating suppression of canonical Wnt signaling under oxidative stress conditions. Treatment with MAPE significantly restored the expression of these genes in a concentration-dependent manner (Figure 6A,B). At the highest concentration (0.02%), *WNT3A* and *LEF1* recovered to approximately 92.5% and 75.9% of the untreated control, respectively, suggesting restoration of Wnt-mediated transcriptional activity. The magnitude of this restoration was comparable to that observed with the well-known antioxidant EGCG (10 µM), supporting the ability of MAPE to modulate this pathway.

In contrast, the Wnt inhibitor *DKK1*, which was strongly upregulated in H_2_O_2_-treated fibroblasts, was significantly downregulated following MAPE treatment (Figure 6C). Since elevated *DKK1* expression is known to inhibit β-catenin signaling and impair dermal regeneration in aged fibroblasts, its suppression implies a release of autocrine inhibition within the Wnt axis. Collectively, these results indicate that MAPE effectively counteracts the oxidative suppression of Wnt signaling by simultaneously upregulating activators (*WNT3A*, *LEF1*) and downregulating the inhibitor (*DKK1*).

### 3.8. MAPE Induces Wnt-Related Transcriptional Responses Comparable to CHIR99021

**Figure 7:**
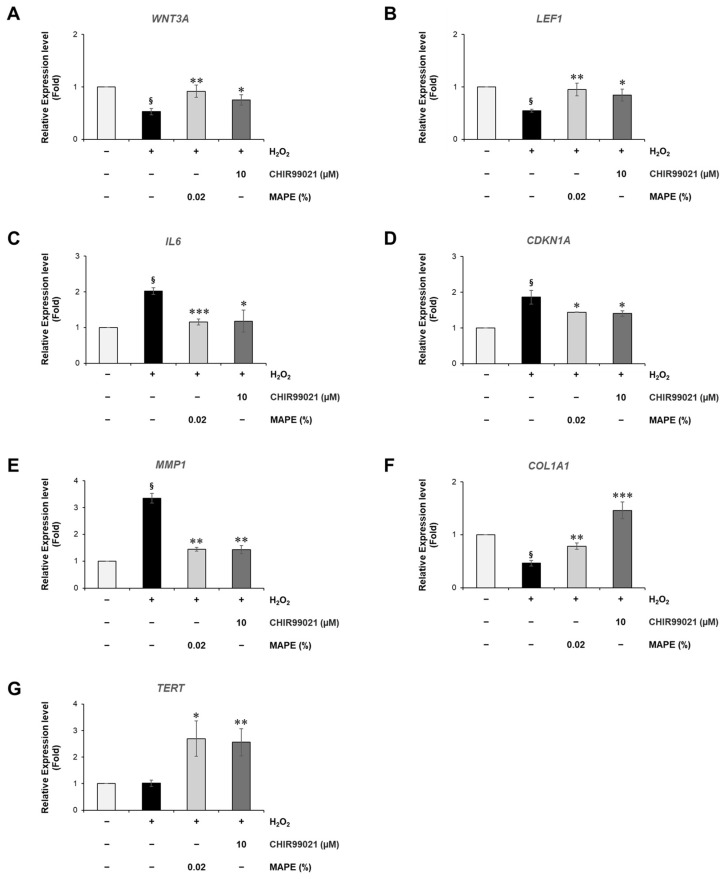
MAPE induces Wnt-related transcriptional responses comparable to the canonical Wnt agonist CHIR99021. HDFs were treated with H_2_O_2_ (300 µM) followed by MAPE (0.02%) or CHIR99021 (10 µM) for 5 days. (**A**) Relative mRNA expression of *WNT3A* and (**B**) *LEF1* determined by qRT-PCR. (**C**) *IL6*, (**D**) *CDKN1A*, and (**E**) *MMP1* expression levels. (**F**) *COL1A1* and (**G**) *TERT* expression levels. Data are presented as mean ± SD (*n* = 3). ^§^ *p* < 0.05 vs. untreated control; * *p* < 0.05, ** *p* < 0.01, *** *p* < 0.001 vs. H_2_O_2_-treated group.

To examine whether the anti-senescent effects of MAPE are associated with modulation of Wnt signaling, we compared its transcriptional responses with those of CHIR99021 (CHIR, 10 µM), a well-established canonical Wnt pathway activator. As expected, CHIR treatment markedly enhanced the expression of *WNT3A* and *LEF1*, two core components of the Wnt/β-catenin signaling cascade (Figure 7A,B). MAPE treatment produced a highly similar pattern, significantly upregulating both genes in H_2_O_2_-stressed fibroblasts. These results indicate that MAPE promotes activation of the canonical Wnt signaling pathway in a manner comparable to the potent Wnt agonist CHIR.

Downstream of Wnt signaling, both CHIR and MAPE suppressed the senescence and inflammatory markers *IL6*, *CDKN1A*, and *MMP1* (Figure 7C–E), which are typically elevated in oxidative stress-induced fibroblast senescence. The reduction in *IL6* and *CDKN1A* suggests inhibition of SASP propagation and cell-cycle arrest, while decreased *MMP1* reflects recovery of ECM integrity.

In parallel, MAPE and CHIR both significantly increased the expression of *COL1A1* and *TERT* (Figure 7F,G), indicating restoration of ECM synthesis and possible reactivation of telomerase-related regenerative capacity. While the effects were broadly comparable, the induction magnitude for *COL1A1* was visibly higher in the CHIR group. These observations support the hypothesis that the anti-senescent effects of MAPE are associated with modulation of the Wnt/β-catenin signaling pathway. This is consistent with previous reports linking Wnt/β-catenin activity to the upregulation of *TERT* and attenuation of cellular senescence [28,29,30].

Collectively, these findings suggest that MAPE may function as a natural modulator of the Wnt signaling pathway, mimicking the regenerative transcriptional profile induced by chemical Wnt activation and linking redox modulation to reactivation of fibroblast regenerative signaling.

### 3.9. MAPE Attenuates Replicative Senescence in Fibroblasts by Suppressing SASP and Restoring ECM and TERT Expression

**Figure 8:**
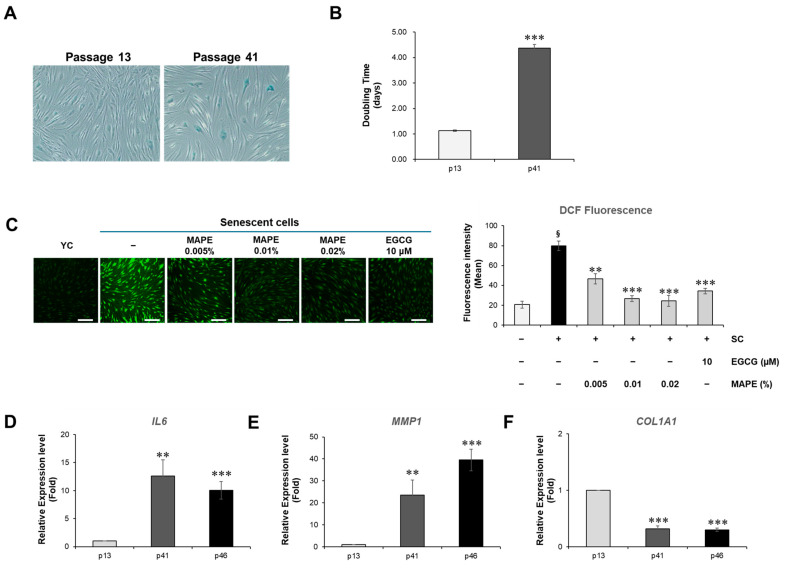
Replicative senescence in HDFs is characterized by SASP upregulation and ECM loss. (**A**) SA-β-gal staining of early-passage (p13) and late-passage (p41) fibroblasts. Late-passage cells show a higher proportion of SA-β-gal-positive (blue) cells with enlarged, flattened morphology, consistent with replicative senescence. (**B**) Population doubling time is markedly prolonged (~4-fold) at p41 relative to p13, indicating reduced proliferative capacity. (**C**) Intracellular ROS levels were assessed by DCF fluorescence in young control cells, senescent cells (SC), and MAPE-treated senescent cells. Representative fluorescence images are shown in the left panel, and the fluorescence intensity is quantified in the right panel. MAPE treatment reduced intracellular ROS levels in senescent cells. Gene expression analysis by qRT-PCR demonstrated a progressive increase in *IL6* (**D**) and *MMP1* (**E**) expression, together with a pronounced reduction in *COL1A1* (**F**), indicating activation of SASP and loss of ECM homeostasis during replicative aging. Data are expressed as mean ± SD (*n* = 3). Statistical significance: ^§^ *p* < 0.05 vs. young control; ** *p* < 0.01, *** *p* < 0.001 vs p13 or adjacent passage groups. Scale bars: 200 µm.

To examine whether MAPE exerts anti-senescent effects under intrinsic, replication-driven aging conditions, we evaluated its activity in late-passage human dermal fibroblasts (passage ≥ 40), a well-established model of replicative senescence. Compared with early-passage fibroblasts (p8–15), aged cells exhibited characteristic senescent morphology—enlarged, flattened cytoplasm with reduced proliferative capacity—and showed strong SA-β-galactosidase positivity along with a 3.87-fold prolongation in population doubling time, confirming a senescent state (Figure 8A,B).

At the molecular level, replicatively senescent fibroblasts demonstrated multiple hallmarks of intrinsic aging. Intracellular ROS levels, quantified by DCFH-DA fluorescence, were markedly elevated, indicating a high oxidative burden characteristic of replication-induced cellular stress (Figure 8C, left panels). In parallel, senescent fibroblasts showed strong upregulation of *IL6* and *MMP1*, accompanied by a notable reduction in *COL1A1* expression, reflecting a SASP-associated pro-inflammatory and ECM-degrading phenotype that contributes to dermal matrix deterioration during intrinsic skin aging (Figure 8D–F).

To exclude the possibility that the observed anti-senescent effects were due to altered cell survival, we evaluated the cytotoxicity of MAPE in replicatively senescent fibroblasts. MAPE did not induce detectable cytotoxicity within the tested concentration range after 5 days of treatment (Supplementary Figure S2).

**Figure 9:**
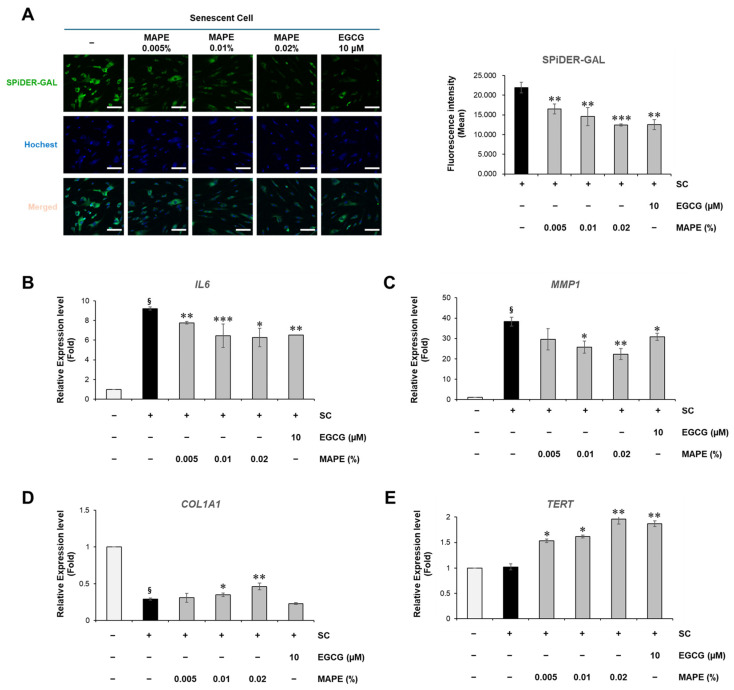
MAPE mitigates replicative fibroblast senescence by suppressing SASP and restoring ECM and TERT expression. (**A**) SPiDER-GAL staining showing SA-β-gal-associated fluorescence in late-passage senescent fibroblasts treated with MAPE (0.005–0.02%) for 5 days. Quantification (**right**) indicates a significant reduction compared with vehicle senescent control (SC). (**B**–**E**) qRT-PCR analysis of senescence- and regeneration-related genes. MAPE decreased *IL6* (**B**) and *MMP1* (**C**) expression in a dose-dependent manner, while *COL1A1* expression was restored (**D**). *TERT* mRNA expression increased following MAPE treatment (**E**). EGCG (10 µM) served as an antioxidant comparator. Data are mean ± SD (*n* = 3). ^§^ *p* < 0.05 vs. young control; * *p* < 0.05, ** *p* < 0.01, *** *p* < 0.001 vs. senescent control. Scale bars: 100 µm.

Treatment with MAPE (0.005–0.02%) for 5 days significantly alleviated these senescent alterations. MAPE reduced intracellular ROS accumulation to levels comparable to the antioxidant reference EGCG (10 µM), indicating effective mitigation of oxidative stress (Figure 8C). Consistent with this reduction in oxidative stress, cellular senescence, assessed by SPiDER-GAL fluorescence, was significantly reduced in a dose-dependent manner following MAPE exposure (Figure 9A). In parallel, MAPE effectively reversed the core senescent molecular hallmarks (Figure 9B–E). *IL6* and *MMP1* expression decreased significantly (Figure 9B,C), while *COL1A1* expression was restored (Figure 9D). Notably, *TERT* mRNA—barely detectable in vehicle senescent controls—was re-induced by MAPE (Figure 9E), suggesting a potential involvement of telomerase-associated mechanisms. The antioxidant comparator EGCG (10 µM) produced similar trends but generally did not exceed the highest MAPE dose.

Taken together, these data indicate that MAPE suppresses SASP (*IL6*/*MMP1*) and reinstates ECM synthesis (*COL1A1*) while reactivating *TERT* in replicatively aged fibroblasts, thereby shifting the cellular state toward a less senescent and more regenerative phenotype.

### 3.10. MAPE Activates Wnt/β-Catenin Signaling in Replicative Senescent Fibroblasts

**Figure 10:**
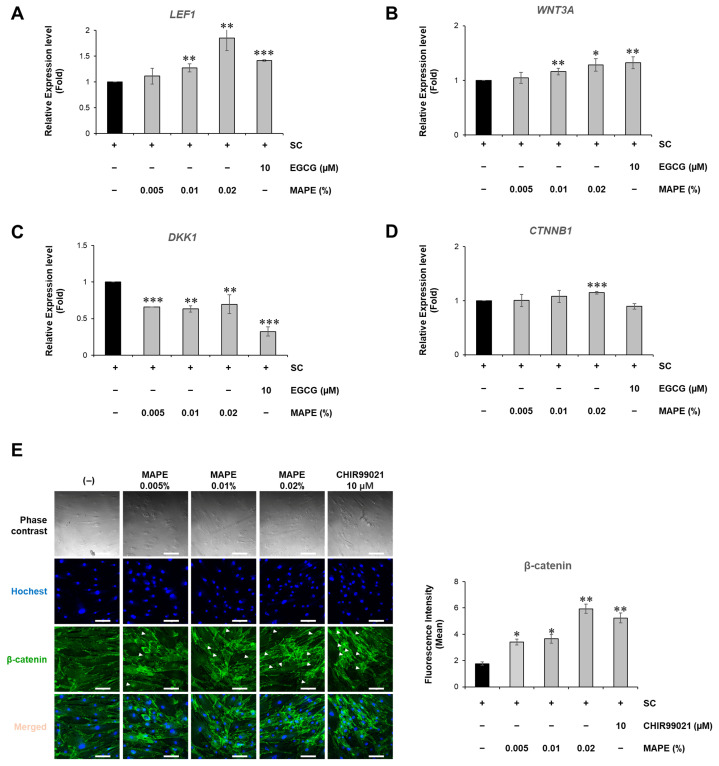
Expression of Wnt signaling-related genes in replicative senescent fibroblasts. (**A**–**D**) Relative mRNA expression levels of Wnt signaling-related genes determined by qRT-PCR: *LEF1* (**A**), *WNT3A* (**B**), *DKK1* (**C**), and *CTNNB1* (**D**). Treatment with MAPE (0.005–0.02%) increased *LEF1* and *WNT3A* expression while decreasing *DKK1* expression. *CTNNB1* expression was moderately increased at 0.02% MAPE. Senescent control (SC) was used as the untreated baseline, and EGCG (10 μM) served as a positive control. (**E**) Representative immunofluorescence images and quantification of β-catenin expression in replicative senescent fibroblasts. Cells were treated with MAPE at the indicated concentrations or CHIR99021 (10 μM) as a positive control. Nuclei were stained with Hoechst (blue), and β-catenin was visualized using a specific antibody (green). Data are presented as mean ± SD (*n* = 3). * *p* < 0.05, ** *p* < 0.01, *** *p* < 0.001 vs. SC. Scale bars: 100 µm.

We investigated whether MAPE could activate Wnt/β-catenin signaling in replicative senescent fibroblasts. qPCR analysis showed that MAPE increased the expression of *LEF1* and *WNT3A* in a dose-dependent manner (Figure 10A,B), while significantly reducing the expression of the Wnt inhibitor *DKK1* (Figure 10C). A significant increase in *CTNNB*1(β-catenin) mRNA expression was also observed at 0.02% MAPE (Figure 10D). These results indicate that MAPE effectively reactivates the canonical Wnt pathway in senescent fibroblasts.

Immunofluorescence staining further supported this activation. MAPE enhanced β-catenin protein levels, and notably promoted its nuclear translocation (indicated by white arrowheads), particularly at 0.02%. This nuclear accumulation was comparable to that induced by CHIR99021, a well-established canonical Wnt activator used as a positive control (Figure 10E). Nuclear accumulation of β-catenin is a hallmark of canonical Wnt pathway activation, consistent with the transcriptional upregulation observed.

Collectively, these findings indicate that MAPE promotes activation of the Wnt/β-catenin signaling pathway in replicative senescent fibroblasts and suggest its potential to mitigate intrinsic cellular aging by enhancing Wnt-driven regenerative pathways.

## 4. Discussion

Skin aging represents a multifactorial process in which oxidative stress, chronic inflammation, and fibroblast senescence converge to drive structural and functional decline of the dermis [31]. Although many antioxidants and botanical extracts have been reported to mitigate certain aspects of skin aging, few natural compounds have been demonstrated to restore both redox balance and pro-regenerative signaling processes associated with fibroblast function.

In parallel with the search for new actives, there is increasing interest in extraction strategies that are compatible with long-term topical use. Polyol solvents such as glycerol and 1,3-butylene glycol are being adopted as water-miscible, low-irritancy “green” alternatives to volatile organic solvents for preparing cosmetic-grade botanical extracts, and they allow the resulting extracts to be incorporated directly into formulations without solvent-removal steps [32,33]. When combined with microwave-assisted extraction, these polyol systems offer additional advantages, including accelerated mass transfer, shorter processing times and reduced thermal degradation of phenolic constituents compared with conventional heating methods [34,35]. In this study, we identified MAPE, a standardized fraction of Jeju-derived propolis prepared by microwave-assisted extraction, as a propolis-derived extract that attenuates fibroblast senescence and supports fibroblast functional recovery, potentially through modulation of Wnt/β-catenin signaling.

One of the key findings is that MAPE not only reduced intracellular ROS but also activated transcriptional antioxidant defenses via upregulation of NRF2 target genes, *NQO1* and *GCLM*. This indicates a mode of action beyond simple radical scavenging. The decline of NRF2 activity with age is a well-established hallmark of skin aging [36,37], and thus the capacity of MAPE to restore this pathway suggests potential for long-term protection against oxidative stress.

In parallel, MAPE suppressed central senescence-associated markers, including SA-β-gal and the senescence-related genes *CDKN2A*, *CDKN1A*, and *IL6*. The reduction in IL-6 is particularly noteworthy because this cytokine is a well-established SASP-associated inflammatory mediator implicated in the propagation of inflammaging during cellular senescence [38,39]. By attenuating SASP, MAPE may not only reduce the senescent phenotype of fibroblasts but also interrupt the paracrine signaling cascade that spreads senescence signals to neighboring skin cells, an outcome that few natural extracts have been shown to achieve.

Another important implication lies in ECM preservation. MAPE prevented *MMP1* upregulation while restoring *COL1A1* expression, a balance essential for maintaining dermal architecture. Impaired collagen turnover is a defining feature of aged skin [40,41], and the ability of MAPE to simultaneously suppress degradation and promote synthesis positions it as a promising matrix-preserving agent. This molecular restoration translated into functional recovery, as validated by wound healing assays where MAPE significantly accelerated fibroblast migration and cellular repair.

A particularly novel aspect of this work is the demonstration that MAPE reactivated the Wnt/β-catenin pathway. Wnt signaling is crucial for fibroblast proliferation, ECM synthesis, and tissue regeneration, yet is progressively lost with age [42,43,44,45]. Restoration of *WNT3A*, *LEF1*, and β-catenin nuclear localization by MAPE, together with suppression of the inhibitor *DKK1*, suggests that this extract may counteract a key molecular driver of dermal aging. The induction of *TERT* expression may indicate involvement of telomerase-related regulatory pathways, although additional studies assessing telomerase activity and telomere dynamics will be necessary to clarify this relationship. This aligns with emerging evidence that Wnt signaling intersects with telomerase regulation and tissue renewal [46]. Furthermore, considering the interplay between redox homeostasis and signaling pathways, the suppression of oxidative stress by MAPE (via NRF2) may create a favorable intracellular environment that facilitates the reactivation of Wnt signaling, suggesting a mutually reinforcing dual mechanism.

To further clarify the mechanism, the canonical Wnt activator CHIR99021 was employed as a positive control. The phenotypic outcomes observed with MAPE—such as reduced *IL6* and *MMP1* and increased *COL1A1*, *LEF1*, and *WNT3A*—closely mirrored those induced by CHIR99021. This concordance indicates that the anti-senescent and repair-supporting effects of MAPE are, at least in part, mediated through modulation of the Wnt/β-catenin signaling pathway.

HPLC profiling of MAPE provided phytochemical context by identifying chrysin, pinocembrin, and galangin as putative active constituents. These flavonoids are consistently reported in propolis [19,47,48], and have been shown to activate NRF2, inhibit NF-κB, and modulate Wnt/β-catenin signaling in various biological systems [49,50,51,52]. In our study, microwave-assisted extraction with butylene glycol yielded the highest total polyphenol content among the tested extraction conditions while preserving representative flavonoid markers at levels comparable to, or slightly higher than, those obtained with high-temperature extraction. Taken together, these findings suggest that the flavonoid constituents present in MAPE may contribute, at least in part, to the coordinated antioxidant and signaling-modulating effects observed in this study. However, because MAPE is a complex natural extract, the relative contribution of each individual compound requires further investigation. In addition, although the chromatographic profiles confirmed the presence of representative flavonoid markers, the biological assays in this study were conducted using a single well-characterized MAPE preparation. Future studies evaluating biological reproducibility across independent extraction batches will therefore be necessary to further establish extract standardization.

Canonical Wnt/β-catenin signaling plays an important physiological role in skin homeostasis, fibroblast function, and tissue repair. In the present study, MAPE modulated Wnt-related gene expression and increased *TERT* mRNA levels under oxidative stress conditions in vitro. These findings should therefore be interpreted as pathway modulation associated with attenuation of cellular senescence rather than sustained proliferative signaling. From a cosmetic perspective, topical exposure and the skin barrier are expected to limit persistent pathway activation. Nevertheless, further studies using advanced skin models and longer-term exposure conditions will be valuable to further characterize the safety profile of MAPE.

Despite these promising findings, this study has limitations. While HPLC identified key flavonoids, direct evidence linking specific individual compounds to Wnt/β-catenin activation requires further investigation. In addition, although the upregulation of canonical NRF2 target genes (*NQO1* and *GCLM*) together with reduced intracellular ROS supports the involvement of NRF2-associated antioxidant signaling, causal validation using pharmacological or genetic inhibition of NRF2 was not performed in this study and should be addressed in future investigations. Likewise, although transcriptional activation of Wnt pathway components and β-catenin nuclear localization support modulation of canonical Wnt signaling, direct pathway-interference experiments such as β-catenin inhibition or knockdown were not performed in this study. Moreover, the observed increase in *TERT* mRNA does not directly demonstrate telomerase activation; further studies assessing telomerase activity and telomere dynamics will be necessary to clarify this mechanism. Finally, as this study was conducted in vitro, future research should validate the efficacy and safety of MAPE in wound healing models, dermal substitutes, or clinical settings to confirm potential benefits for skin repair.

Therefore, this study contributes to the growing body of evidence supporting the anti-aging potential of propolis and its derivatives. However, unlike previous reports that primarily emphasized antioxidant or anti-inflammatory effects [14,15,16,17,18], our findings extend these insights by demonstrating coordinated modulation of NRF2 and Wnt/β-catenin–TERT signaling in fibroblast senescence models. This dual mechanism provides a compelling rationale for further evaluation of MAPE in ex vivo human skin equivalents and in vivo applications.

## 5. Conclusions

MAPE, a standardized extract of Jeju propolis prepared by microwave-assisted extraction, combines a distinct botanical origin with an optimized extraction process, resulting in improved bioactive properties. In this study, MAPE alleviated oxidative stress, limited fibroblast senescence, preserved ECM integrity, and modulated senescence-related signaling pathways in human dermal fibroblasts. These findings suggest that MAPE may represent a promising cosmeceutical ingredient for mitigating skin aging and supporting skin repair-related processes.

## Figures and Tables

**Table 1:** Primer sequences used for qRT-PCR analysis.

Gene Name	Accession No.	Primer	Sequence (5′→3′)
*CDKN2A*	NM_058195	Forward	GGGTTTTCGTGGTTCACATCC
		Reverse	CTAGACGCTGGCTCCTCAGTA
*CDKN1A*	NM_000389	Forward	AGGTGGACCTGGAGACTCTCAG
		Reverse	TCCTCTTGGAGAAGATCAGCCG
*IL6*	NM_000600	Forward	AGACAGCCACTCACCTCTTCAG
		Reverse	TTCTGCCAGTGCCTCTTTGCTG
*NQO1*	NM_000903	Forward	CCTGCCATTCTGAAAGGCTGGT
		Reverse	GTGGTGATGGAAAGCACTGCCT
*GCLM*	NM_002061	Forward	CATTTACAGCCTTACTGGGAGG
		Reverse	ATGCAGTCAAATCTGGTGGCA
*GCLC*	NM_001498	Forward	GGAAGTGGATGTGGACACCAGA
		Reverse	GCTTGTAGTCAGGATGGTTTGCG
*MMP1*	NM_002421	Forward	AAAATTACACGCCAGATTTGCC
		Reverse	GGTGTGACATTACTCCAGAGTTG
*COL1A1*	NM_000088	Forward	ATCAACCGGAGGAATTTCCGT
		Reverse	CACCAGGACGACCAGGTTTTC
*WNT3A*	NM_033131	Forward	ATGAACCGCCACAACAACGAGG
		Reverse	GTCCTTGAGGAAGTCACCGATG
*DKK1*	NM_012242	Forward	GGTATTCCAGAAGAACCACCTTG
		Reverse	CTTGGACCAGAAGTGTCTAGCAC
*LEF1*	NM_016269	Forward	AGAACACCCCGATGACGGA
		Reverse	GGCATCATTATGTACCCGGAAT
*TERT*	NM_198253	Forward	GCCGATTGTGAACATGGACTACG
		Reverse	GCTCGTAGTTGAGCACGCTGAA
*CTNNB1*	NM_001098209	Forward	CACAAGCAGAGTGCTGAAGGTG
		Reverse	GATTCCTGAGAGTCCAAAGACAG

## Data Availability

The original contributions presented in this study are included in the article. Further inquiries can be directed to the corresponding author.

## Supplementary Materials

The following supporting information can be downloaded at: https://www.mdpi.com/article/10.3390/antiox15030395/s1, Table S1: Screening of 20 common phenolic compounds in MAPE by HPLC analysis; Figure S1: Comparison of HPLC chromatograms of 20 phenolic standards and MAPE; Figure S2: Effects of MAPE on cell viability in replicatively senescent human dermal fibroblasts.

## Abbreviations

The following abbreviations are used in this manuscript:

CHIR	CHIR99021
ECM	Extracellular matrix
GAE	Gallic acid equivalents
HDFs	Human dermal fibroblasts
HPLC	High-performance liquid chromatography
MAE	Microwave-assisted extraction
MAPE	Microwave-assisted propolis extract
PDA	Photodiode array
PDT	Population doubling time
SASP	Senescence-associated secretory phenotype
TPC	Total phenolic content

## References

[1] López-Otín C., Blasco M.A., Partridge L., Serrano M., Kroemer G. (2013). The hallmarks of aging. Cell.

[2] Quan T., Fisher G.J. (2015). Role of age-associated alterations of the dermal extracellular matrix microenvironment in human skin aging: A mini-review. Gerontology.

[3] Zhang J., Zhang R., Ding C., Sun Y., Wang Z. (2024). Aging in the Dermis: Fibroblast Senescence and its significance. Aging Cell.

[4] Finkel T., Holbrook N.J. (2000). Oxidants, oxidative stress and the biology of ageing. Nature.

[5] Tanigawa S., Fujii M., Hou D.-X. (2007). Action of Nrf2 and Keap1 in ARE-mediated NQO1 expression by quercetin. Free Radic. Biol. Med..

[6] Ma Q. (2013). Role of Nrf2 in oxidative stress and toxicity. Annu. Rev. Pharmacol. Toxicol..

[7] Zhang H., Davies K.J.A., Forman H.J. (2015). Oxidative Stress Response and Nrf2 Signaling in Aging. Free Radic. Biol. Med..

[8] Phan T.G., Holland J.D. (2020). Lef1 Expression in Fibroblasts Enhances Skin Repair and Reduces Scarring. eLife.

[9] Lim X., Nusse R. (2013). Wnt signaling in skin development, homeostasis, and disease. Cold Spring Harb. Perspect. Biol..

[10] Shi Y., Wang F., Xiang L., Chen Y. (2015). Wnt and Notch Signaling Pathway Involved in Wound Repair: DKK1 Treatment Delays Healing. Stem Cell Res. Ther..

[11] Sarkar A., Fisher P.B. (2020). Wnt signaling in telomerase regulation and its relevance to tissue regeneration. Aging Dis..

[12] Pasupuleti V.R., Sammugam L., Ramesh N., Gan R.Y. (2017). Honey, propolis, and royal jelly: A comprehensive review of their biological actions and health benefits. Oxid. Med. Cell. Longev..

[13] Ristori F., Lazzeri A., Neri I., D’Amico A., Cini N. (2020). Propolis: A natural source of antioxidants. Antioxidants.

[14] Murase H., Shimazawa M., Kakino M., Ichihara K., Tsuruma K., Hara H. (2013). The effects of Brazilian green propolis against excessive light-induced cell damage in retina and fibroblast cells. Evid.-Based Complement. Altern. Med..

[15] Bae I.A., Ha J.W., Choi J.Y., Boo Y.C. (2022). Antioxidant effects of Korean propolis in HaCaT keratinocytes exposed to particulate matter 10. Antioxidants.

[16] Koga H., Nishimura T., Kobayashi K. (2025). Effects of Brazilian green propolis and artepillin C on collagen metabolism and fibroblast behaviors: Implications for skin wound healing. Phytomedicine.

[17] Marquele F.D., Xavier C.R., Vizioli N.L., Fonseca Y.M., Fonseca M.J. (2008). Evaluation of the potential of Brazilian propolis against UV-induced oxidative stress. Evid.-Based Complement. Alternat. Med..

[18] Kim S.-K., Han S.M., Kim S.G., Kim H.Y., Ryu S., Woo S.O. (2021). The mechanism of anti-inflammation effects of propolis components in Raw264.7 macrophage cell. J. Apic..

[19] Xu W., Lu H., Yuan Y., Deng Z., Zheng L., Li H. (2022). The antioxidant and anti-inflammatory effects of flavonoids from propolis via Nrf2 and NF-κB pathways. Foods.

[20] Proestos C., Komaitis M. (2008). Application of Microwave-Assisted Extraction to the Fast Extraction of Plant Phenolic Compounds. LWT—Food Sci. Technol..

[21] Chan C.-H., Yusoff R., Ngoh G.-C., Kung F.W.-L. (2011). Microwave-Assisted Extractions of Active Ingredients from Plants. J. Chromatogr. A.

[22] Touzani S., Imtara H., Katekhaye S., Mecchate H., Ouassou H., Alqahtani A.S., Noman O.M., Nasr F.A., Fearnley H., Fearnley J. (2021). Determination of Phenolic Compounds in Various Propolis Samples Collected from an African and an Asian Region and Their Impact on Antioxidant and Antibacterial Activities. Molecules.

[23] Lee J.-J., Ng S.-C., Hsu J.-Y., Liu H., Chen C.-J., Huang C.-Y., Kuo W.-W. (2022). Galangin Reverses H_2_O_2_-Induced Dermal Fibroblast Senescence via SIRT1–PGC-1α/Nrf2 Signaling. Int. J. Mol. Sci..

[24] Talachi N., Afzal E., Nouri M., Abroun S., Zarrabi M., Jahandar H. (2023). Protective Effect of Human Amniotic Membrane Extract against Hydrogen Peroxide-Induced Oxidative Damage in Human Dermal Fibroblasts. Int. J. Cosmet. Sci..

[25] Elder R.L. (1985). Final report on the safety assessment of butylene glycol, hexylene glycol, ethoxydiglycol, and dipropylene glycol. J. Am. Coll. Toxicol..

[26] Coppé J.-P., Patil C.K., Rodier F., Sun Y., Muñoz D.P., Goldstein J., Nelson P.S., Desprez P.-Y., Campisi J. (2008). Senescence-associated secretory phenotypes reveal cell-nonautonomous functions of oncogenic RAS and the p53 tumor suppressor. PLoS Biol..

[27] Coppé J.-P., Desprez P.-Y., Krtolica A., Campisi J. (2010). The senescence-associated secretory phenotype: The dark side of tumor suppression. Annu. Rev. Pathol..

[28] Cai Y., Sun H., Song X., Zhao J., Xu D., Liu M. (2023). The Wnt/β-catenin signaling pathway inhibits osteoporosis by regulating the expression of TERT: An in vivo and in vitro study. Aging.

[29] Bauer K., Tao S., Rudolph K.L. (2013). Telomere stability—Wnt it or lose it. EMBO Rep..

[30] Lehmann J., Narcisi R., Franceschini N., Chatzivasileiou D., Boer C.G., Koevoet W.J.L.M., Putavet D., Drabek D., van Haperen R., de Keizer P.L.J. (2022). WNT/beta-catenin signalling interrupts a senescence-induction cascade in human mesenchymal stem cells that restricts their expansion. Cell. Mol. Life Sci..

[31] Qin Z., Voorhees J.J., Fisher G.J., Quan T. (2014). Age-associated reduction of cellular spreading/mechanical force up-regulates matrix metalloproteinase-1 expression and collagen fibril fragmentation via c-Jun/AP-1 in human dermal fibroblasts. Aging Cell.

[32] Myo H., Yaowiwat N., Pongkorpsakol P., Aonbangkhen C., Khat-Udomkiri N. (2023). Butylene glycol used as a sustainable solvent for extracting bioactive compounds from *Camellia sinensis* flowers with ultrasound-assisted extraction. ACS Omega.

[33] Roselli V., Pugliese G., Leuci R., Brunetti L., Gambacorta L., Tufarelli V., Piemontese L. (2024). Green Methods to Recover Bioactive Compounds from Food Industry Waste: A Sustainable Practice from the Perspective of the Circular Economy. Molecules.

[34] Win S.M., Saelee M., Myo H., Khat-Udomkiri N. (2024). Microwave-assisted extraction of phenolic compounds and antioxidants for cosmetic applications using polyol-based technology. J. Vis. Exp..

[35] Khat-udomkiri N., Win S.M. (2025). Microwave-assisted butylene glycol extraction: An environmentally friendly method for isolating bioactive compounds from coffee silverskin with antioxidant, anti-tyrosinase, and anti-melanogenic effects. Ind. Crops Prod..

[36] Medoro A., Saso L., Scapagnini G., Davinelli S. (2023). NRF2 signaling pathway and telomere length in aging and age-related diseases. Mol. Cell. Biochem..

[37] Buttari B., Tramutola A., Rojo A.I., Chondrogianni N., Saha S., Berry A., Giona L., Miranda J.P., Profumo E., Davinelli S. (2025). Proteostasis decline and redox imbalance in age-related diseases: The therapeutic potential of NRF2. Biomolecules.

[38] Franceschi C., Campisi J. (2014). Chronic inflammation (inflammaging) and its potential contribution to age-associated diseases. J. Gerontol. A Biol. Sci. Med. Sci..

[39] Freund A., Orjalo A.V., Desprez P.-Y., Campisi J. (2010). Inflammatory networks during cellular senescence: Causes and consequences. Trends Mol. Med..

[40] Fisher G.J., Varani J., Voorhees J.J. (2008). Looking older: Fibroblast collapse and therapeutic implications. Arch. Dermatol..

[41] Fisher G.J., Quan T., Purohit T., Shao Y., Cho M.K., He T., Varani J., Kang S., Voorhees J.J. (2009). Collagen fragmentation promotes oxidative stress and elevates matrix metalloproteinase-1 in fibroblasts in aged human skin. Am. J. Pathol..

[42] Liu J., Xiao Q., Xiao J., Niu C., Li Y., Zhang X., Zhou Z., Shu G., Yin G. (2022). Wnt/β-catenin signalling: Function, biological mechanisms, and therapeutic opportunities. Signal Transduct. Target. Ther..

[43] Xue C., Chu Q., Shi Q., Zeng Y., Lu J., Li L. (2025). Wnt signaling pathways in biology and disease: Mechanisms and therapeutic advances. Signal Transduct. Target. Ther..

[44] Wang X., Zhu Y., Sun C., Wang T., Shen Y., Cai W., Sun J., Chi L., Wang H., Song N. (2017). Feedback activation of basic fibroblast growth factor signaling via the Wnt/β-catenin pathway in skin fibroblasts. Front. Pharmacol..

[45] Makrantonaki E., Brink T.C., Zampeli V., Elewa R.M., Mlody B., Hossini A.M., Hermes B., Krause U., Knolle J., Abdallah M. (2012). Identification of biomarkers of human skin ageing in both genders. Wnt signalling—A label of skin ageing?. PLoS ONE.

[46] Gu B.-W., Apicella M., Mills J.A., Fan J.-M., Reeves D.A., French D.L., Podsakoff G.M., Bessler M., Mason P.J. (2015). Impaired telomere maintenance and decreased canonical WNT signaling but normal ribosome biogenesis in induced pluripotent stem cells from X-linked dyskeratosis congenita patients. PLoS ONE.

[47] Barbarić M., Mišković K., Bojić M., Baus Lončar M., Smolčić-Bubalo A., Debeljak Ž., Medić-Šarić M. (2011). Chemical composition of the ethanolic propolis extracts and its effect on HeLa cells. J. Ethnopharmacol..

[48] Touzani S., Embaslat W., Imtara H., Kmail A., Kadan S., Zaid H., ElArabi I., Badiaa L., Saad B. (2019). In vitro evaluation of the potential use of propolis as a multitarget therapeutic product: Physicochemical properties, chemical composition, and immunomodulatory, antibacterial, and anticancer properties. Biomed Res. Int..

[49] Oršolić N., Jazvinšćak Jembrek M. (2022). Molecular and cellular mechanisms of propolis and its polyphenolic compounds against cancer. Int. J. Mol. Sci..

[50] Habtemariam S. (2019). The Nrf2/HO-1 axis as targets for flavanones: Neuroprotection by pinocembrin, naringenin, and eriodictyol. Oxid. Med. Cell. Longev..

[51] Li J., Peng X.-L., Cheng X.-Y., Yang J.-J., Cui C., Liu J.-H. (2025). Pinocembrin Alleviates Postoperative Cognitive Dysfunction in Aged Mice by Modulating miR-384-5p/FZD1 Axis to Activate the Wnt/β-Catenin Pathway. Mol. Neurobiol..

[52] Wu C., Chen J., Chen C., Wang W., Wen L., Gao K., Chen X., Xiong S., Zhao H., Li S. (2015). Wnt/β-Catenin Coupled with HIF-1α/VEGF Signaling Pathways Involved in Galangin Neurovascular Unit Protection from Focal Cerebral Ischemia. Sci. Rep..

