# Supplementary material for: Microwave-Assisted Propolis Extract Attenuates Oxidative-Stress- and Replicative Senescence via NRF2 and Wnt/β-Catenin–TERT Activation in Human Dermal Fibroblasts
Source: Antioxidants (Basel). 2026 Mar 20;15(3):395. doi: 10.3390/antiox15030395 (PMC13024501; doi:10.3390/antiox15030395)
Supplement: Supplementary file 1 [file antioxidants-15-00395-s001.zip › antioxidants-4174551-supplementary.pdf]

Supplementary Table S1. Screening of 20 common phenolic compounds in MAPE by HPLC analysis.

No.	Compound name	R.T.(min)	Result
1	Gallic acid	4.8	ND
2	D-(-)-Salicin	7.6	ND
3	Protocatechuic acid	8.9	ND
4	Scopolin	13.5	ND
5	Chlorogenic acid	14.3	ND
6	Puerarin	16.4	ND
7	Caffeic acid	17.2	ND
8	Vanillin	21.6	ND
9	*p*-Coumaric acid	23.0	ND
10	Ferulic acid	23.5	ND
11	Rutin Hydrate	24.8	ND
12	Narirutin	25.5	ND
13	Hesperidin	27.1	ND
14	Rosmarinic acid	30.0	ND
15	Myricetin	30.7	ND
16	Quercetin	37.2	ND
17	(±)-Naringenin	41.4	ND
18	Apigenin	42.2	ND
19	Kaempferol	43.1	ND
20	Formononetin	46.6	ND

Supplementary Figure S1. Comparison of HPLC chromatograms of 20 phenolic standards and MAPE.

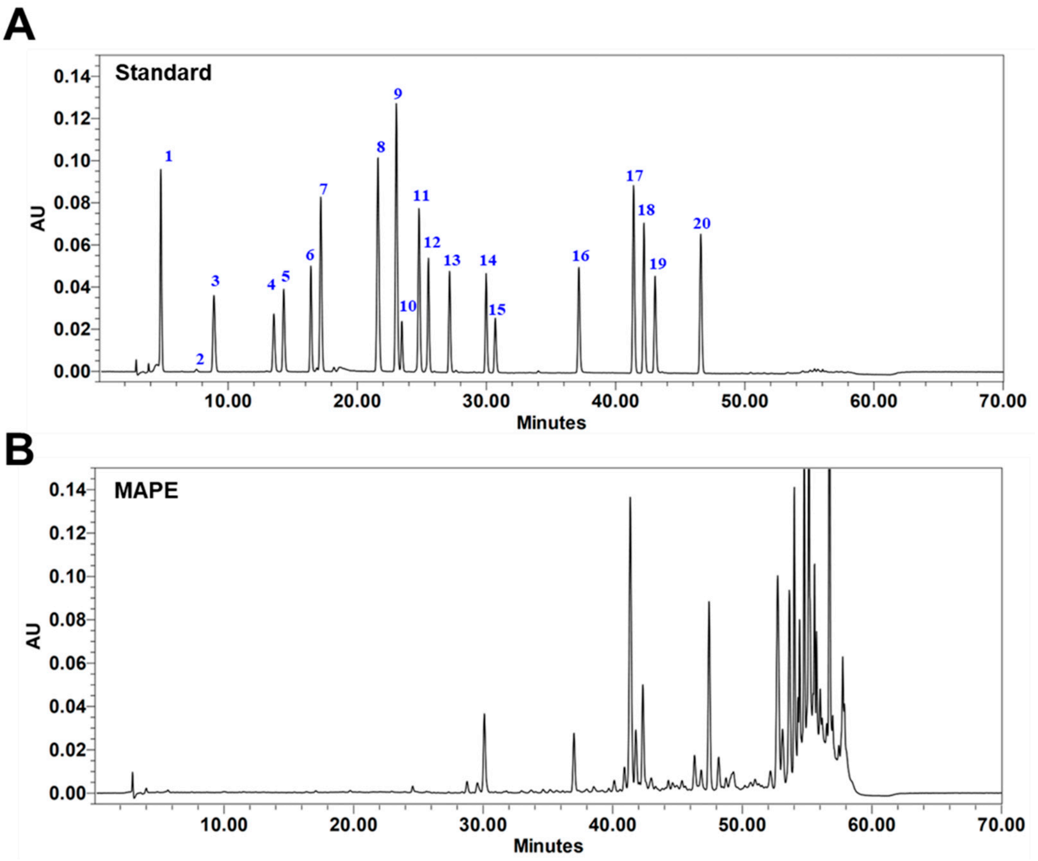

Figure S1. (A) Representative HPLC chromatogram of 20 phenolic standard compounds. The numbers above the peaks correspond to the compounds listed in Supplementary Table S1. (B) Representative HPLC chromatogram of MAPE analyzed under the same chromatographic conditions. The 20 phenolic standards were not detected in MAPE under these analytical conditions.

Supplementary Figure S2. Effects of MAPE on cell viability in replicatively senescent human dermal fibro blasts**.**

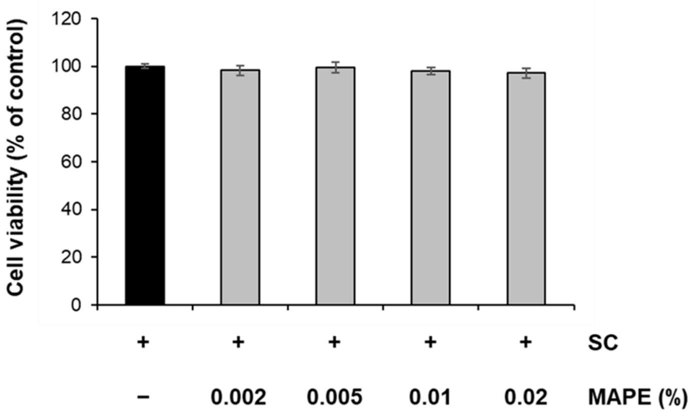

Figure S2. Replicatively senescent human dermal fibroblasts were treated with MAPE at the indicated concentrations (0.002–0.02%) for 5 days. Cell viability was measured to evaluate potential cytotoxic effects of MAPE. No detectable cytotoxicity was observed across the tested concentration range. Data in are presented as mean ± SD (n = 4).
